# Incidental Lung Lesions on the CT Component of Oncologic FDG PET/CT: A Pictorial Review of Interpretive Pitfalls and Diagnostic Clues

**DOI:** 10.3390/diagnostics16182910

**Published:** 2026-09-09

**Authors:** Narae Lee, Hyukjin Yoon, Ie Ryung Yoo

**Affiliations:** 1 Division of Nuclear Medicine, Department of Radiology, St. Vincent’s Hospital, College of Medicine, The Catholic University of Korea, 222 Banpo-daero, Seocho-gu, Seoul 06591, Republic of Korea; lnr84@catholic.ac.kr (N.L.); atznawa@gmail.com (H.Y.); 2 Division of Nuclear Medicine, Department of Radiology, Seoul St. Mary’s Hospital, College of Medicine, The Catholic University of Korea, 222 Banpo-daero, Seocho-gu, Seoul 06591, Republic of Korea

**Keywords:** incidental lung lesion, pulmonary nodule, FDG PET/CT, differential diagnosis, pulmonary metastasis, primary lung cancer, oncologic imaging

## Abstract

^18^F-fluorodeoxyglucose (FDG) positron emission tomography (PET)/computed tomography (CT) is widely used for oncologic staging, restaging, and response assessment. Its CT component frequently reveals pulmonary lesions that are not the principal target of the examination. In patients with known malignancy, such lesions may have a higher pre-test probability of malignancy than incidental pulmonary lesions detected in the general population and cannot be reliably characterized by CT morphology or FDG uptake alone. Small lesion size, partial-volume effects, respiratory motion, and low tumor cellularity may render metastases metabolically occult, whereas infectious and inflammatory processes may show intense FDG uptake and mimic malignancy. This review presents a case-based, lesion-by-lesion approach integrating thin-section CT morphology, concordance or discordance between FDG uptake and CT findings, interval evolution, and oncologic and clinical context. Illustrative cases demonstrate PET-occult pulmonary metastases, inflammatory mimics, coexisting infection and metastasis, synchronous or metachronous primary lung cancer, and subsolid nodules representing either atypical metastases or primary lung adenocarcinoma. Subsolid or cavitary morphology, waxing-and-waning interval changes, and low FDG uptake should not prompt premature benign labeling. Systematic evaluation of the CT component, followed by lesion-based integration of imaging and clinical data, is essential for characterizing these lesions and may help reduce both false-positive and false-negative interpretations.

## 1. Introduction

^18^F-fluorodeoxyglucose (FDG) PET/CT is a standard procedure for staging, restaging, and monitoring therapeutic response in various malignancies [1,2]. Although the primary role of FDG PET/CT is metabolic assessment, its CT component—often performed without intravenous contrast or using a low-dose protocol—frequently reveals pulmonary abnormalities that are not the principal target of the examination. Such findings may be newly detected on the CT images or may be clinically significant despite being inconspicuous on the metabolic images. In this review, we refer to these findings as incidental lung lesions (ILLs). In a series of patients with non-small cell lung cancer undergoing integrated PET/CT, clinically relevant abnormalities lacking FDG uptake were frequently identified on the CT component alone [3].

ILLs in patients with known malignancy require a different interpretive approach from nodules detected in otherwise healthy screening populations. The Fleischner Society recommendations explicitly exclude patients with known primary cancers at risk for metastasis [4]. To date, no dedicated consensus guideline addresses incidentally detected pulmonary nodules in patients with known malignancy. In the absence of such guidance, a survey of thoracic radiologists found that management of these nodules relied largely on individual experience [5]. In that survey, most respondents reported all incidentally detected nodules and routinely recommended follow-up CT. An AJR Expert Panel review emphasizes individualized risk assessment in oncologic patients, considering metastasis, second primary lung cancer, infection, treatment-related change, postoperative or post-radiation change, and benign scarring [6]. That review addresses the broader management of incidentally detected pulmonary nodules in oncologic patients, largely in the context of dedicated diagnostic CT. The present article instead focuses on interpretive challenges specific to the CT component of FDG PET/CT, where morphologic and metabolic information must be integrated within a single examination. These challenges include discordance between FDG uptake and CT findings, protocol-related constraints of routine noncontrast low-dose acquisition, and respiratory misregistration between PET and CT.

FDG uptake alone is often insufficient for reliable lesion characterization. The inherent limitations of the PET technique necessitate a meticulous, integrated diagnostic strategy. Small lesion size, partial-volume effects, respiratory motion, and low tumor cellularity may result in little or no FDG uptake despite the presence of malignancy. Conversely, inflammatory and infectious processes can demonstrate intense FDG accumulation and mimic tumor activity. Consequently, reliance on PET findings without careful CT analysis may lead to both false-negative and false-positive interpretations.

The CT component of PET/CT therefore plays a central role in evaluating ILLs. Interpretation should integrate thin-section CT morphology, concordance or discordance between FDG uptake and CT findings, interval evolution, and the patient’s oncologic and clinical context. Accordingly, a systematic lesion-by-lesion approach is essential. This case-based review illustrates that framework with representative cases to reduce false-negative interpretations of PET-occult malignancy and false-positive interpretations arising from inflammatory or infectious mimics.

**Literature Search.** A literature search was conducted in PubMed/MEDLINE through August 2026 using combinations of terms related to “pulmonary nodule,” “incidental pulmonary nodule,” “pulmonary metastasis,” “lung cancer,” “ground-glass nodule,” “FDG PET/CT,” “oncologic imaging,” “cancer patient,” “inflammation,” “infection,” “tuberculosis,” “nontuberculous mycobacteria,” “post-treatment change,” “partial-volume effect,” and “respiratory motion.” Searches were conducted iteratively using Boolean combinations of the listed terms. In general, (“FDG PET/CT” OR “oncologic imaging”) was combined with one or more lesion-, disease-, mimic-, or technical-factor terms using AND, while related terms within each concept were combined with OR. Reference lists of relevant reviews, society guidelines, expert panel statements, and key original studies were also screened for additional publications. Articles were selected based on their relevance to the imaging characteristics, differential diagnosis, temporal evolution, or management of pulmonary lesions encountered on oncologic FDG PET/CT. Recent literature was preferentially incorporated where available, while earlier studies were retained when they represented key evidence for specific morphologic, metabolic, or clinical observations that had not been superseded by more recent data.

**Scope and Case Selection.** This article is a narrative, case-based pictorial review rather than a systematic review. The recurrent interpretive pitfalls addressed in this review were defined and contextualized through the literature search described above. Representative cases were then purposively chosen from oncologic FDG PET/CT examinations performed at Seoul St. Mary’s Hospital to illustrate these pitfalls. The cases were selected solely for educational illustration and do not constitute a consecutive clinical series. No estimates of prevalence, diagnostic performance, or clinical outcomes are derived from them. Final diagnoses were established by histopathology whenever available, supplemented by clinicopathologic correlation where required. In one case without histopathologic confirmation, the diagnosis was based on disease-specific imaging and biochemical findings with 2.5 years of imaging follow-up. The basis for the final diagnosis of each case is specified in the corresponding figure legend.

## 2. Mechanistic and Technical Basis of Interpretive Error

Interpretive errors involving ILLs often arise from predictable properties of the tracer, lesion, and acquisition protocol. These mechanisms are outlined before the individual clinical scenarios are presented.

### 2.1. Why Some Malignancies Show Low FDG Uptake

The magnitude of FDG accumulation in a lesion reflects both viable tumor cell density per unit volume and the glycolytic activity of those cells, the latter influenced by the expression of glucose transporters and hexokinase. In primary non-small cell lung cancer, glucose transporter type 1 (GLUT-1) expression correlates with maximum standardized uptake value (SUVmax) [7,8]. Similar associations between FDG uptake and molecular markers have also been reported in metastatic pulmonary tumors [9]. Squamous cell carcinoma generally demonstrates higher GLUT-1 expression and SUVmax than adenocarcinoma [7,8,10]. Within adenocarcinoma, solid-predominant tumors show significantly higher SUVmax than those with other predominant histologic patterns. GLUT-1 expression is likewise higher in tumors with a solid growth pattern than in those without [11].

Two mechanisms therefore explain why a malignant lesion may show little or no FDG uptake. First, low cellular density: in lepidic-predominant adenocarcinoma and ground-glass nodules (GGNs), neoplastic cells grow along intact alveolar walls while the airspaces remain aerated, so the number of metabolically active cells within any given voxel is small. In invasive mucinous adenocarcinoma, abundant extracellular mucin further dilutes the cellular component. Second, low intrinsic glycolytic activity: GLUT-1 expression is lower in tumors lacking a solid growth pattern [11]. In subsolid nodules and in tumors with a mucinous component, absent uptake therefore carries little negative predictive value.

### 2.2. Why Benign Processes Show High FDG Uptake

FDG is a marker of glucose metabolism rather than of malignancy, and activated inflammatory cells are avid consumers of glucose. Neutrophils, activated macrophages, and granulation tissue upregulate glucose transporters during acute inflammation and subsequent tissue repair, and the resulting FDG uptake may equal or exceed that of many tumors [12]. This is the metabolic basis for false-positive interpretations in granulomatous disease, organizing pneumonia, mycobacterial infection, and post-treatment inflammatory change [13]. Because the underlying biology is shared, no SUV threshold reliably separates inflammatory from neoplastic uptake; differentiation must therefore rely on CT morphology, distribution, temporal behavior, and clinical context rather than on metabolic intensity alone.

Clinical context also includes regional epidemiology. In settings where tuberculosis (TB) and nontuberculous mycobacterial (NTM) pulmonary disease remain prevalent, granulomatous disease is an important cause of unexpected FDG-avid pulmonary nodules. Active tuberculomas may demonstrate uptake indistinguishable from that of pulmonary metastases or primary lung cancer, and sarcoidosis, fungal infection, and organizing pneumonia may produce a similar metabolic pattern [13]. NTM infection may likewise present as consolidation with intense uptake, with a reported SUVmax of 26.9 in an immunocompetent patient in whom biopsy showed only caseating granulomas [14]. Regional prevalence therefore modifies the pre-test probabilities of competing diagnoses and increases the importance of morphologic assessment.

Certain morphologic patterns may help narrow the differential. Bilateral small nodules, branching centrilobular (tree-in-bud) nodules, and cylindrical bronchiectasis are among the most common thin-section CT findings of NTM pulmonary disease [15]. Their presence favors an airway-centered infectious process. These patterns may be subtle on the low-dose CT component and are best assessed on thin-section lung-window reconstructions. Even against this infectious background, a lesion that progressively enlarges or otherwise differs from the surrounding abnormalities in morphology or temporal behavior should be evaluated independently, as discussed in Section 5.

### 2.3. Quantitative Limits of SUVmax

The widely cited SUVmax threshold of 2.5 for characterizing a pulmonary nodule as malignant is an empirical convention rather than a biologically derived cutoff. Its performance degrades in precisely the situations encountered in oncologic surveillance. The dominant technical constraint is the partial-volume effect, which arises from the finite spatial resolution of clinical PET systems and produces progressive underestimation of SUV as lesion volume decreases [16]. The magnitude is substantial: a lesion measuring approximately 6 mm in its shortest axis may have its SUVmax underestimated by 60–70%, and full recovery of the true value is not approached until the lesion measures roughly 18 mm in minor axis or more [17]. Respiratory motion further compounds partial-volume effects by blurring pulmonary lesions, which reduces the measured SUV and increases the apparent lesion volume, particularly in the middle and lower portions of the lungs [18].

Two practical points follow: First, a subcentimeter pulmonary nodule with SUVmax below 2.5 should not be considered metabolically inactive; rather, the low SUV may simply reflect its small size. Correction approaches using lesion size measured on CT have been proposed for this reason [19]. Second, SUVmax is not a transferable number: it varies with scanner model, reconstruction algorithm, iteration and subset settings, use of point-spread-function modeling and time-of-flight, uptake time, blood glucose level, and multiple patient- and acquisition-related factors [16,18,20]. Comparison of SUVmax across institutions or across scanners is therefore unreliable, whereas comparison of serial studies acquired on the same system with the same protocol is more informative. Even then, a change in lesion size alters the degree of partial-volume underestimation, so a fall in SUVmax accompanying tumor shrinkage may reflect geometry rather than a true decline in metabolic rate [17]. In surveillance, the change in uptake—interpreted together with the change in lesion size—is more meaningful than any single absolute value.

### 2.4. Protocol-Inherent Constraints of the CT Component

Detector technology and reconstruction algorithms have narrowed the gap between the CT component of PET/CT and dedicated chest CT, and diagnostic-quality thin-section reconstructions may improve morphologic assessment when available. Two constraints, however, are inherent to the PET/CT protocol rather than to image quality, and are therefore not resolved by improvements in hardware. The examination is usually performed without intravenous contrast material, which precludes assessment of enhancement and limits evaluation of mediastinal and hilar structures. More importantly, PET data are acquired over several minutes during free breathing, whereas CT is captured within seconds at a single respiratory phase [21].

Free-breathing acquisition introduces two major sources of interpretive error. Respiratory motion blurs lesion margins and degrades detection, an effect most pronounced in the lower lobes and juxtadiaphragmatic regions [22]. In addition, mismatch of respiratory phase between the CT and PET datasets produces misregistration, which is most common adjacent to the diaphragm and heart; a curvilinear cold artifact at the lung base and apparent displacement of hepatic dome activity into the lung are the characteristic manifestations, and the latter may simulate a pulmonary lesion [21]. Deep-inspiration breath-hold PET/CT reduces both misregistration and motion-related SUV underestimation, but is not routinely available [23].

The practical implication is not that the CT component should be distrusted, but that it should be read together with the PET data and with prior studies. Any focus of FDG uptake near the diaphragm requires anatomic correlation on the CT component before being assigned to the lung. When the two datasets appear discrepant in this region, misregistration should be considered before the finding is interpreted as true metabolic discordance. The CT component of PET/CT should not be regarded as a substitute for dedicated diagnostic chest CT when intravenous contrast enhancement, comprehensive lung characterization, or detailed mediastinal or hilar assessment is required.

## 3. PET-Occult Pulmonary Metastases on the CT Component

A major pitfall in oncologic PET/CT is dismissing a small pulmonary nodule because it lacks visible FDG uptake. Although the CT component was traditionally acquired at low dose with relatively thick sections, contemporary PET/CT systems incorporate multidetector-row CT and permit thin-section reconstruction. Adding computer-aided detection to thin-section lung reconstruction in the routine PET/CT read-out protocol increases the detection of pulmonary nodules [24]. Deep-learning image reconstruction has also improved nodule detection and measurement accuracy on ultra-low-dose chest CT, suggesting potential applicability to the CT component of PET/CT [25]. Consequently, small pulmonary nodules without corresponding FDG uptake are detected more often on oncologic PET/CT and pose a recurring diagnostic challenge [6,26].

Importantly, incidental pulmonary nodules detected in oncology patients may have a higher pre-test probability of malignancy than those detected in the general population. However, the actual risk varies according to the index malignancy, lesion characteristics, treatment status, and competing infectious or inflammatory diagnoses. Using thin-section CT, Hanamiya et al. detected one or more non-calcified pulmonary nodules in 75% (233/308) of patients with extrapulmonary malignancy and reported that nodules 10 mm or larger and those farther from the pleura were more likely to be malignant [27]. Reported malignancy rates among such nodules vary widely with patient selection and nodule criteria, from about one in five subcentimeter nodules with little or no FDG uptake [28] to 42% in a cohort of patients with extrapulmonary cancers and non-calcified pulmonary nodules [29]. In this clinical setting, nodular pulmonary abnormalities may represent hematogenous metastases, perilymphatic nodularity from lymphangitic spread, or a synchronous primary lung cancer. Therefore, even small discrete pulmonary nodules identified on CT warrant careful attention and follow-up, particularly in patients with biologically aggressive malignancies or known metastatic disease.

**Figure 1:**
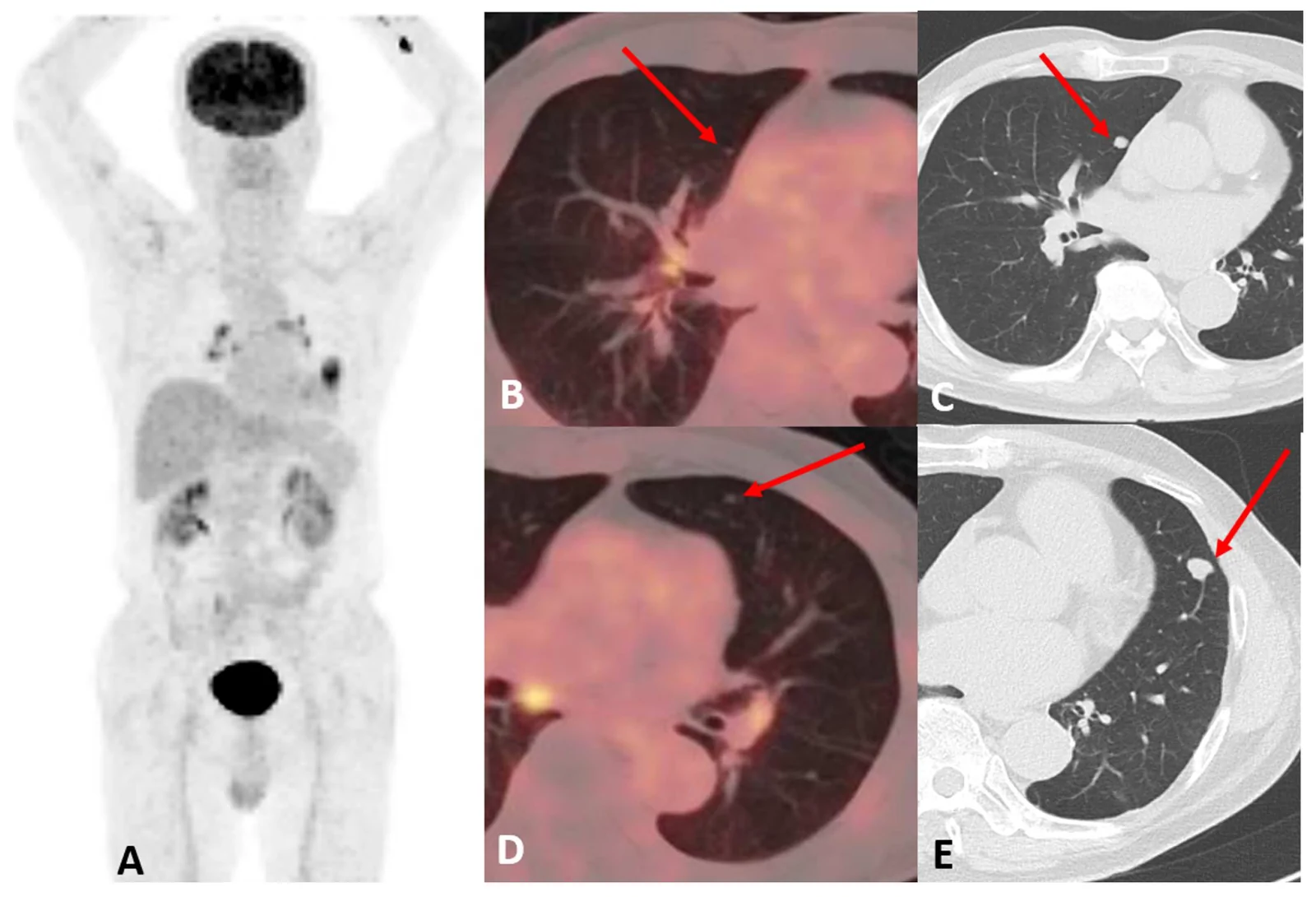
FDG PET-occult pulmonary metastases detected on CT. A man with left lower-lobe non-small cell lung cancer. Small pulmonary nodules (arrows) are identified on the lung window image of the CT component but demonstrate no discernible FDG uptake on the corresponding FDG PET/CT images (**A**,**B**,**D**). Follow-up CT images (**C**,**E**) obtained 1 year later showed interval enlargement of the nodules, and histopathologic examination of a biopsy specimen confirmed metastatic disease.

However, PET itself has inherent technical limitations in the evaluation of small pulmonary lesions. PET negativity does not reliably exclude malignancy in subcentimeter pulmonary nodules because of partial-volume averaging, respiratory motion, and limited viable tumor volume. Respiratory gating has been shown to recover lesion size and tracer-uptake measurements that motion otherwise degrades [30]. In addition, tumors with low cellularity, mucinous components, or indolent biology may show low metabolic activity despite being malignant. O et al. evaluated pulmonary nodules measuring 1 cm or smaller with little or no FDG uptake in patients with nonthoracic malignancies and found that 24 of 121 patients had malignant pulmonary nodules, most likely representing pulmonary metastases, corresponding to a malignancy rate of 19.8%. Notably, malignancy rates were not negligible even when FDG uptake was imperceptible. In that series, the malignancy rate was higher when no other benign-appearing lesion was present elsewhere in the lungs (24.7%) than when one was present (8.3%; adjusted odds ratio 4.9 on logistic regression, *p* = 0.019), whereas nodule multiplicity and FDG-uptake perceptibility were not significant discriminators [28]. Figure 1 illustrates a small pulmonary metastasis that remained metabolically occult on PET but demonstrated interval enlargement on follow-up CT and was subsequently confirmed pathologically.

**Figure 2:**
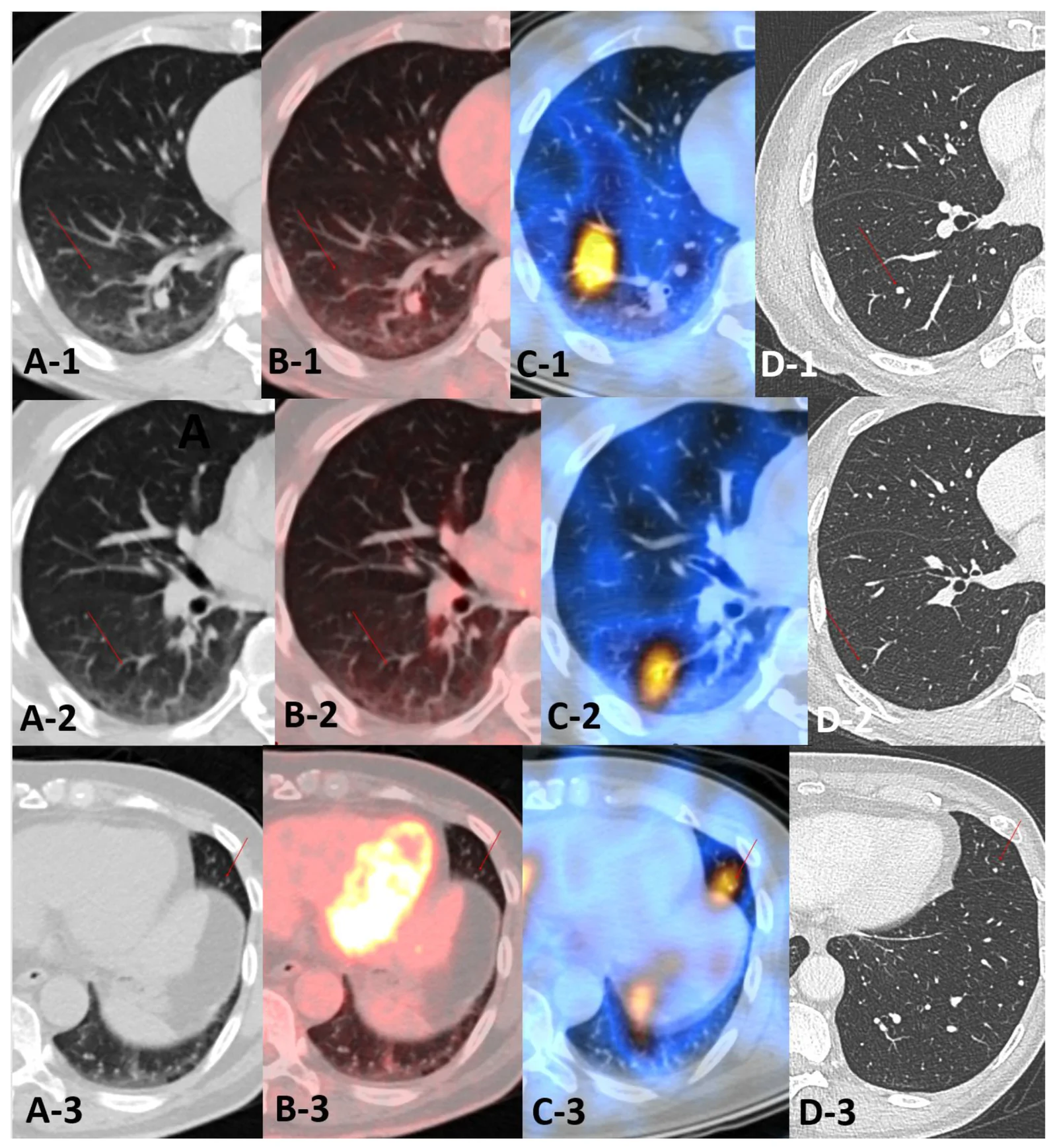
FDG-negative, iodine-avid pulmonary metastases from differentiated thyroid carcinoma. A man treated with total thyroidectomy for papillary thyroid carcinoma was evaluated for a rising serum thyroglobulin level. Each row shows a separate pulmonary nodule (arrows). (**A-1**–**A-3**) The CT component of the FDG PET/CT demonstrated tiny nodules for which (**B-1**–**B-3**) showed no corresponding FDG uptake on the fused images. (**C-1**–**C-3**) Post-therapy SPECT/CT performed 3 months later, following administration of 7.4 GBq (200 mCi) of ^131^I, showed radioiodine accumulation in the same nodules. (**D-1**–**D-3**) Follow-up CT 2.5 years after the initial PET/CT demonstrated slow interval growth, accompanied by progressive elevation of serum thyroglobulin. In the absence of histopathologic confirmation, the diagnosis was based on concordant radioiodine uptake and longitudinal imaging and biochemical changes.

Metabolic silence may also reflect tumor biology and tracer choice rather than lesion size alone. In well-differentiated thyroid carcinoma, iodine avidity and glucose metabolism tend to vary inversely—the so-called flip-flop phenomenon—so that iodine-concentrating metastases are frequently FDG-negative, whereas dedifferentiated deposits lose iodine avidity and become FDG-avid [31]. In a series of 86 patients treated with radioiodine for pulmonary metastases, lesions with greater FDG uptake showed absent iodine accumulation significantly more often, and the reciprocal pattern was most pronounced in older patients [32]. Figure 2 illustrates this pattern in a patient investigated for a rising thyroglobulin level: tiny bilateral pulmonary nodules were visible on the CT component but showed no FDG uptake, and their metastatic nature was supported by radioiodine accumulation on post-therapy imaging and by subsequent growth. When the primary malignancy is inherently poorly FDG-avid, absent FDG uptake has limited diagnostic value, and the CT component may become the primary imaging evidence of disease.

Teaching Point: A PET-negative nodule is not necessarily benign; absent uptake may reflect small size, low cellularity, or limited avidity for the tracer used. Any nodule that persists or enlarges warrants scrutiny regardless of uptake.

## 4. Inflammatory and Metastatic Pulmonary Lesions That Mimic Each Other

At the level of an individual ILL, inflammatory and metastatic pulmonary lesions may mimic each other because neither CT morphology nor FDG uptake reliably distinguishes the two. As outlined in Section 2.2, FDG uptake is not cancer-specific, and inflammatory conditions, including infection, granulomatous disease, radiation pneumonitis, and postoperative change, may show intense FDG accumulation [12,13].

**Figure 3:**
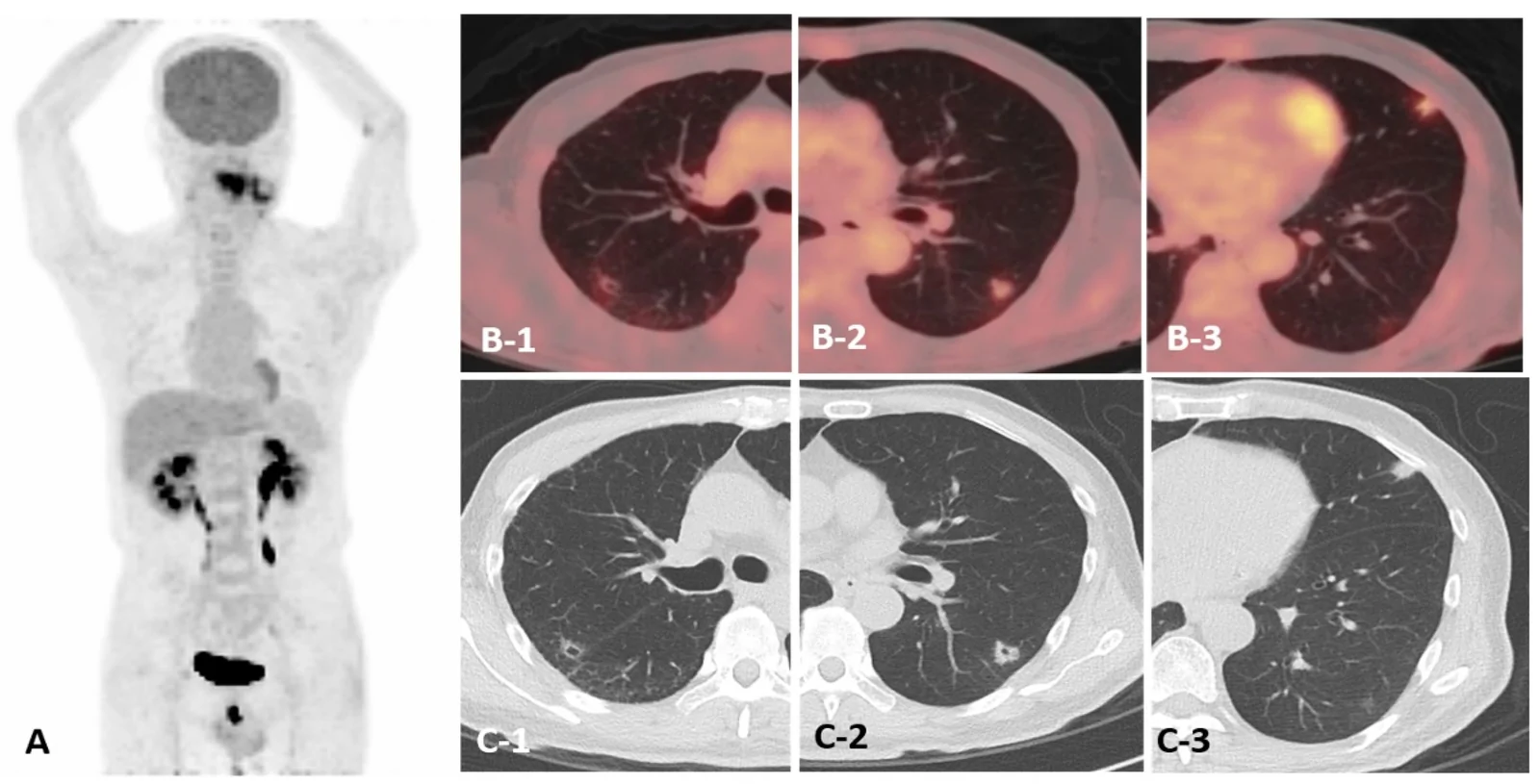
Cavitary/subsolid metastatic nodules mimicking infection. Atypical pulmonary metastases in a man with tongue cancer resemble septic emboli or fungal infection. The FDG PET/CT maximum-intensity projection (**A**), fusion images (**B-1**–**B-3**), and CT images (**C-1**–**C-3**) reveal multiple cavitary, irregular, or subsolid nodules with mild FDG uptake. Wedge resection of the pulmonary nodules confirmed metastatic squamous cell carcinoma.

In addition to metabolic overlap, inflammatory and metastatic lesions may share similar morphologic features on CT. Certain inflammatory patterns, including cavitary nodules, patchy consolidative or subsolid opacities, and airway-centered nodules, may resemble atypical pulmonary metastases. Conversely, several malignancies—including head and neck squamous cell carcinoma, gastrointestinal malignancies, melanoma, and sarcoma—may produce atypical pulmonary metastases with cavitary, necrotic, or inflammatory-appearing morphology [33]. Accordingly, neither intense FDG uptake nor inflammatory-appearing morphology can definitively distinguish benign from malignant lesions [13,33]. Figure 3 illustrates metastatic pulmonary lesions showing both FDG uptake and imaging features suggestive of inflammation.

**Figure 4:**
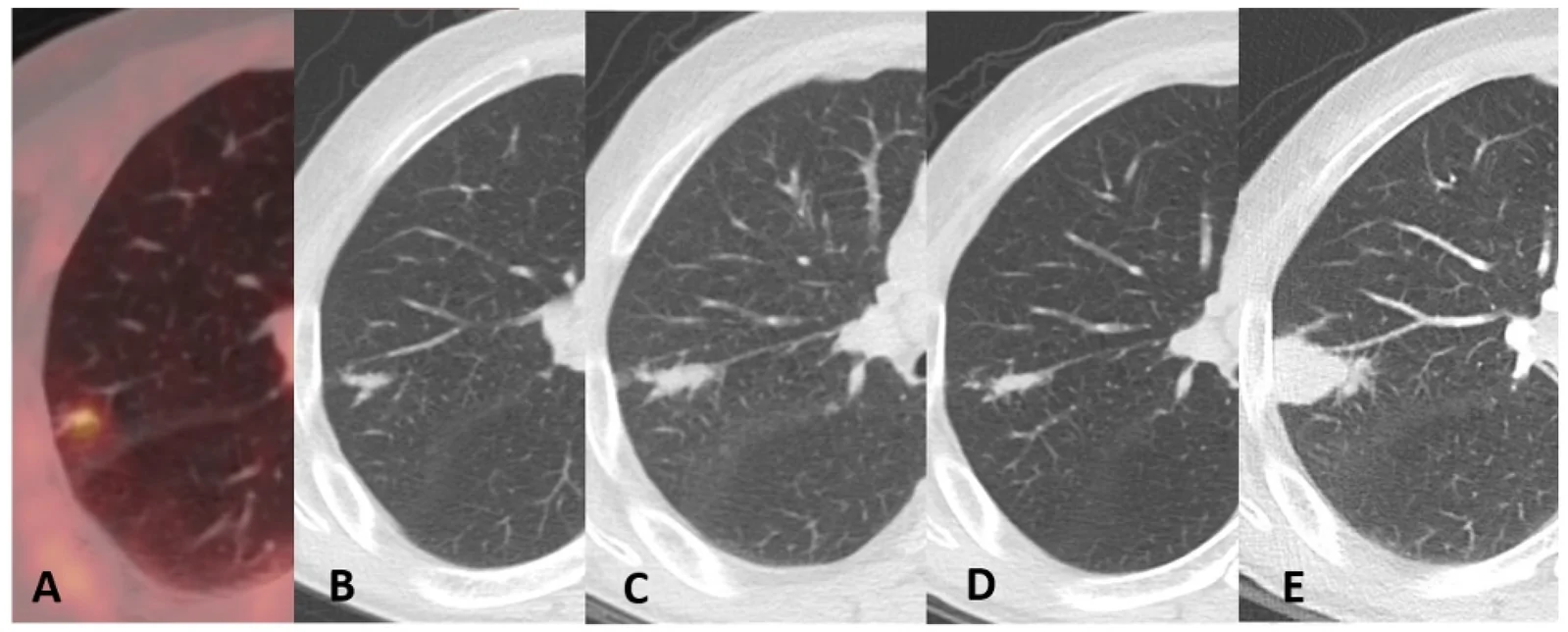
An inflammatory-appearing nodule later confirmed as malignancy. A man with tonsil cancer and lymph node recurrence. (**A**) FDG PET/CT fusion image revealed an irregular branching nodular opacity, leading to CT follow-up. The nodule gradually increased in size on follow-up CT obtained at 5 months (**B**) and 10 months (**C**) after the index PET/CT, and slightly decreased at 11 months (**D**). This transient decrease was initially interpreted as inflammatory; however, the nodule showed substantial growth at 22 months (**E**), and subsequent right upper lobectomy provided histopathologic confirmation of metastatic disease.

Short-term interval decrease may favor inflammation. However, transient regression does not completely exclude malignancy, particularly when the lesion persists, later regrows, or occurs in the setting of a biologically aggressive cancer. In such cases, serial CT review should focus on wall thickening, an increasing solid component, changes in contour, and evolution discordant with that of coexisting inflammatory lesions. Continued imaging follow-up may therefore be warranted, as illustrated in Figure 4.

Teaching Point: Inflammatory and metastatic pulmonary lesions may mimic each other in both morphology and metabolic activity, and transient regression does not exclude malignancy. Serial CT review and integration of clinical context should guide interpretation rather than any single imaging feature.

## 5. Coexistence of Infection and Malignancy

Another important pitfall is assuming that all ILLs within an individual patient share the same etiology. In oncologic patients, metastatic nodules, primary lung cancer, drug-related pneumonitis, aspiration, sequelae of prior granulomatous disease, active TB, NTM pulmonary disease, and other pulmonary infections may coexist [6]. This possibility is especially relevant in patients who have received chemotherapy, immunosuppressive therapy, or repeated antibiotic treatment and in regions where TB and NTM pulmonary disease are prevalent [15].

**Figure 5:**
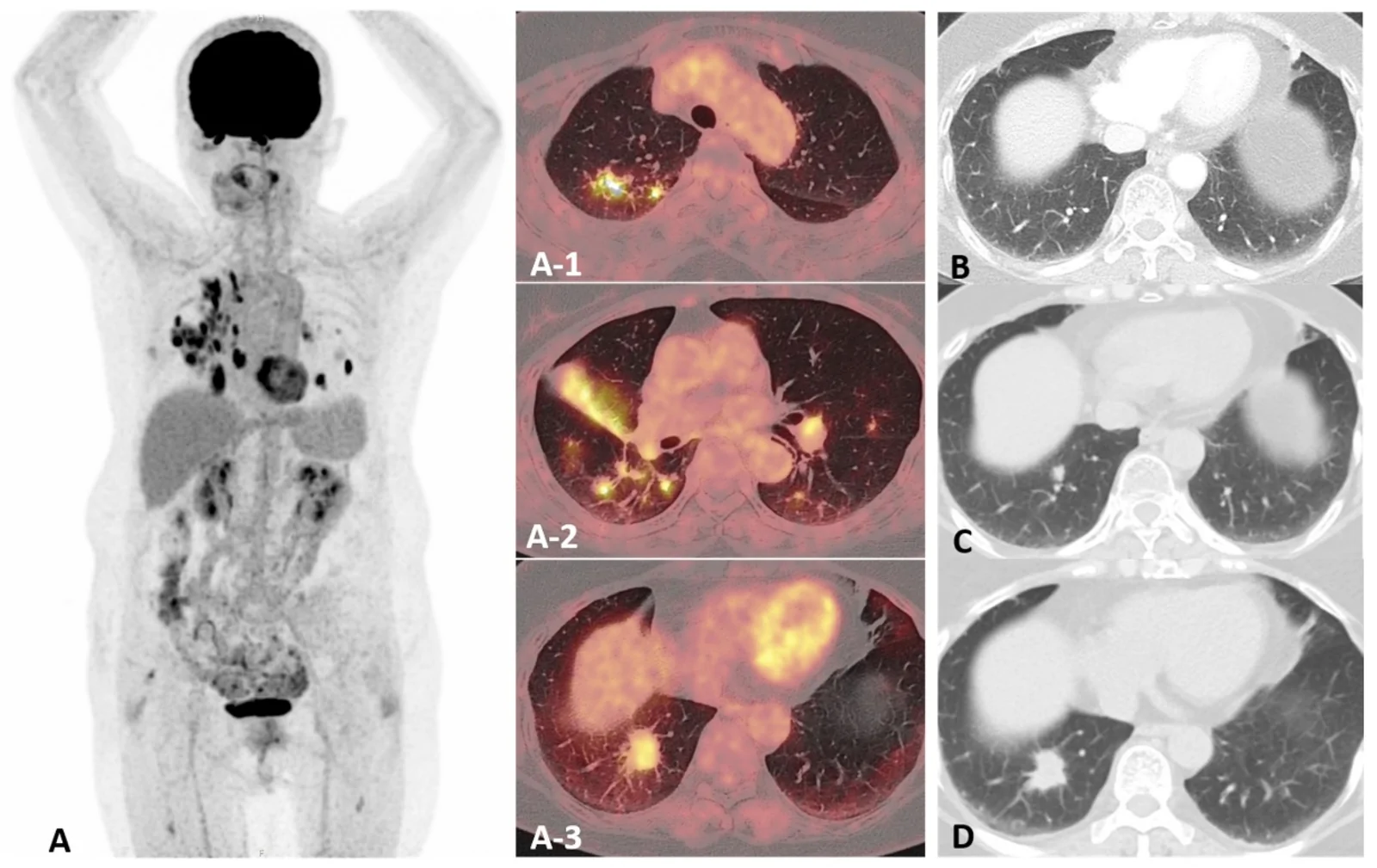
Metastatic cholangiocarcinoma coexisting with nontuberculous mycobacterial (NTM) infection. A woman with a history of common bile duct cancer 9 years earlier underwent FDG PET/CT (**A**,**A-1**–**A-3**) for multiple lung lesions. Prior CT scans obtained 4 years (**B**), 2 years (**C**), and 2 months (**D**) before the index FDG PET/CT were reviewed. The progressively enlarging right lower lobe mass was surgically resected and confirmed as adenocarcinoma, with clinicopathologic findings favoring pulmonary metastasis from the previous cholangiocarcinoma. The other resected lesions showed chronic granulomatous inflammation with multinucleated giant cells and necrosis, and tissue AFB culture grew nontuberculous mycobacteria, confirming NTM infection.

Therefore, the diagnostic unit should be the individual lesion rather than the patient as a whole. Each lesion should be assessed separately for distribution, margins, internal density, cavitation, relationship to the airways, FDG uptake intensity, and temporal evolution. A single progressively enlarging lesion among multiple stable or waxing-and-waning inflammatory nodules should not be dismissed as part of a diffuse infectious process. Conversely, labeling all lesions as metastases may result in overstaging and the loss of potentially curative local treatment options. A representative case of pulmonary metastasis coexisting with NTM pulmonary disease is shown in Figure 5.

Teaching Point: Mixed etiologies may coexist within a single patient with cancer. Lesion-by-lesion longitudinal assessment is essential.

## 6. Synchronous or Metachronous Primary Lung Cancer

In patients with a history of malignancy, a newly detected ILL is often presumed to represent metastatic or recurrent disease. However, a solitary enlarging pulmonary nodule may represent a synchronous or metachronous primary lung cancer, particularly when it shows irregular or spiculated margins, subsolid morphology, pleural retraction, an air bronchogram, or growth independent of the index cancer [6,34]. Correctly identifying a new primary lung cancer is clinically important because potentially curative local therapy may be indicated, whereas metastatic or recurrent disease generally alters staging and directs management toward systemic therapy.

The likelihood that a new pulmonary nodule represents a primary lung cancer rather than a metastasis varies according to the type of the index cancer. In a series of 149 patients with an extrapulmonary malignancy and a solitary pulmonary nodule [35], primary lung cancer was more common than metastasis in patients with head and neck, bladder, breast, cervical, bile duct, esophageal, ovarian, prostate, or gastric carcinoma. Among patients with head and neck cancer, for example, the ratio of primary lung cancers to pulmonary metastases was 25:3. In patients with lymphoma or leukemia, every malignant solitary nodule in that series proved to be a primary lung cancer; none represented pulmonary involvement by the index hematologic malignancy. In contrast, patients with melanoma, sarcoma, or testicular carcinoma were more likely to have a solitary pulmonary metastasis. Smokers had approximately 3.5-fold higher odds of having primary lung cancer than nonsmokers [35].

These estimates pertain specifically to solitary pulmonary nodules in patients with extrapulmonary malignancies. When multiple nodules are present, as in the cavitary metastases from head and neck cancer described in Section 4, metastatic disease becomes more likely, particularly when the lesions show similar morphology and temporal behavior or coexist with other metastases. However, multiplicity alone is not diagnostic, and lesion number and distribution should be considered in conjunction with the biologic behavior and expected metastatic pattern of the index cancer.

When the index cancer is itself a primary lung cancer, the estimates above do not apply. A new pulmonary nodule may represent either recurrent disease, including intrapulmonary metastasis, or a second primary lung cancer, and the distinction requires integration of clinical, radiologic, pathologic, and molecular information. Recurrence is most common during the first two years after curative treatment, but a new solitary pulmonary nodule, particularly after a longer disease-free interval, should also raise consideration of a second primary lung cancer [6]. In this setting, tumors with different histologic types are conventionally classified as separate primaries. When the tumors share the same histology, traditional clinicopathologic criteria have supported a diagnosis of second primary malignancy when the new lesion arises at least two years after the first, originates from carcinoma in situ, or occurs in a different lobe or lung without common lymphatic involvement or distant metastases [6]. Comprehensive histologic and molecular comparison may nevertheless be required when the distinction remains uncertain.

**Figure 6:**
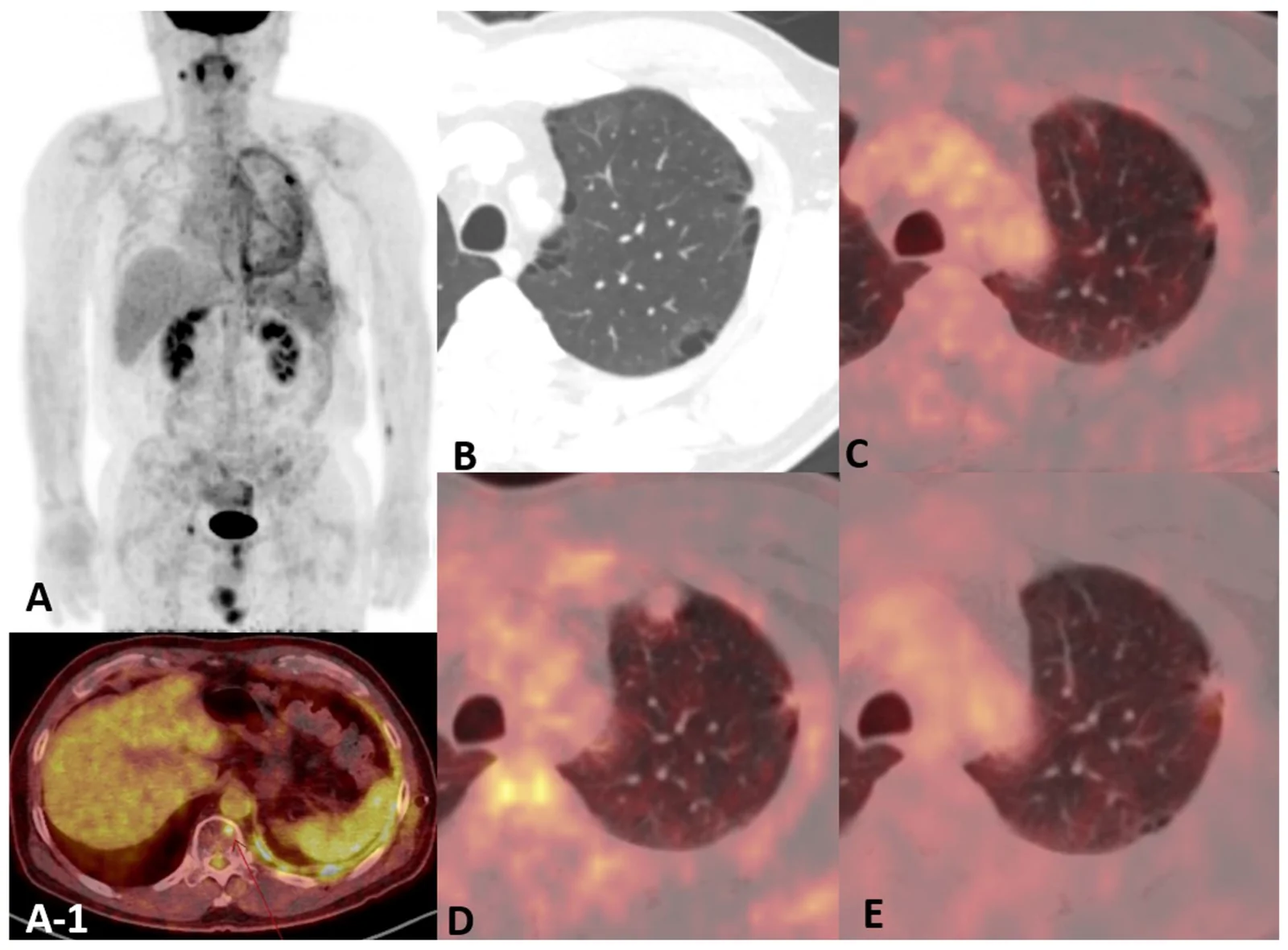
Metachronous primary lung cancer. A man with a history of lymphoma and gastric cancer presented with dyspnea. (**A**,**A-1**) FDG PET/CT revealed FDG-avid left pleural lesions with pleural effusion, along with an FDG-avid T11 vertebral metastasis (red arrow). Retrospective review of prior CT images obtained approximately 15 months (**B**), 14 months (**C**), 12 months (**D**), and 8 months (**E**) before the index FDG PET/CT demonstrated progressive enlargement of a subpleural nodule in the left upper lobe. Histopathologic examination of the left upper lobe wedge resection specimen demonstrated poorly differentiated adenocarcinoma with widespread pleural metastases. Clinicopathologic correlation supported a primary lung cancer.

The possibility of a new primary lung cancer should be explicitly considered when the lesion is solitary, shows the morphologic features outlined above, or is discordant with the expected metastatic or recurrent pattern of the index cancer. Review of prior CT images is essential because slow but persistent growth over months to years may favor a primary lung cancer over rapidly progressive hematogenous metastasis or recurrence. When a new primary lung cancer is considered likely, the subsequent evaluation differs from that for metastatic or recurrent disease. Dedicated thoracic staging, including invasive mediastinal staging when indicated, and tissue confirmation may be needed before curative-intent local therapy. A representative case of metachronous primary lung cancer is shown in Figure 6.

Teaching Point: A new pulmonary nodule in a patient with prior cancer should not be presumed to represent metastatic or recurrent disease. A synchronous or metachronous primary lung cancer should also be considered.

## 7. Subsolid Nodules: Atypical Metastasis Versus Primary Lung Adenocarcinoma

Subsolid ILLs pose a distinct diagnostic challenge in oncologic patients. Low cellular density, small lesion size, and partial-volume effects may limit FDG uptake in both lung adenocarcinoma-spectrum lesions and atypical metastases. Accordingly, neither subsolid morphology nor low FDG uptake establishes benignity or reliably distinguishes an indolent primary lung adenocarcinoma from metastatic disease.

In the general population, an incidentally detected, persistent GGN most often falls within the adenocarcinoma spectrum—from atypical adenomatous hyperplasia (AAH) and adenocarcinoma in situ (AIS) to minimally invasive adenocarcinoma (MIA) and lepidic-predominant invasive adenocarcinoma—rather than representing metastasis [4,36]. For such nodules, dedicated surveillance strategies exist. According to the Fleischner Society recommendations, a solitary pure GGN ≥ 6 mm should undergo follow-up CT at 6–12 months to confirm persistence and then every 2 years until 5 years, whereas a part-solid nodule measuring ≥6 mm should be reassessed at 3–6 months and, if unchanged with the solid component remaining <6 mm, followed annually for 5 years [4]. As noted in Section 1, however, these recommendations were not developed for patients at risk for pulmonary metastases. Therefore, management in patients with cancer cannot rely solely on the typically indolent course of adenocarcinoma-spectrum GGNs and must incorporate the patient’s oncologic context.

In oncologic patients, three features may help weigh the likelihood of a primary lung adenocarcinoma versus a subsolid metastasis. Multiplicity favors metastasis, particularly when other metastatic lesions are present and behave concordantly, whereas a solitary persistent lesion favors a primary adenocarcinoma. The identity of the index cancer also matters, because GGN-forming or other alveolar-pattern metastases have been documented in melanoma and pancreatic adenocarcinoma. A subsolid nodule in a patient whose index cancer is not known to metastasize in this pattern therefore favors a new lung primary. Growth rate may provide a third clue: in the cases illustrated here, the metastatic subsolid lesion enlarged over about a year, whereas the primary adenocarcinoma evolved over several years. None of these features is decisive in isolation, and FDG uptake may initially be absent or faint in both; the assessment therefore rests primarily on the CT component, integrating multiplicity, interval evolution, and the metastatic behavior of the index cancer.

**Figure 7:**
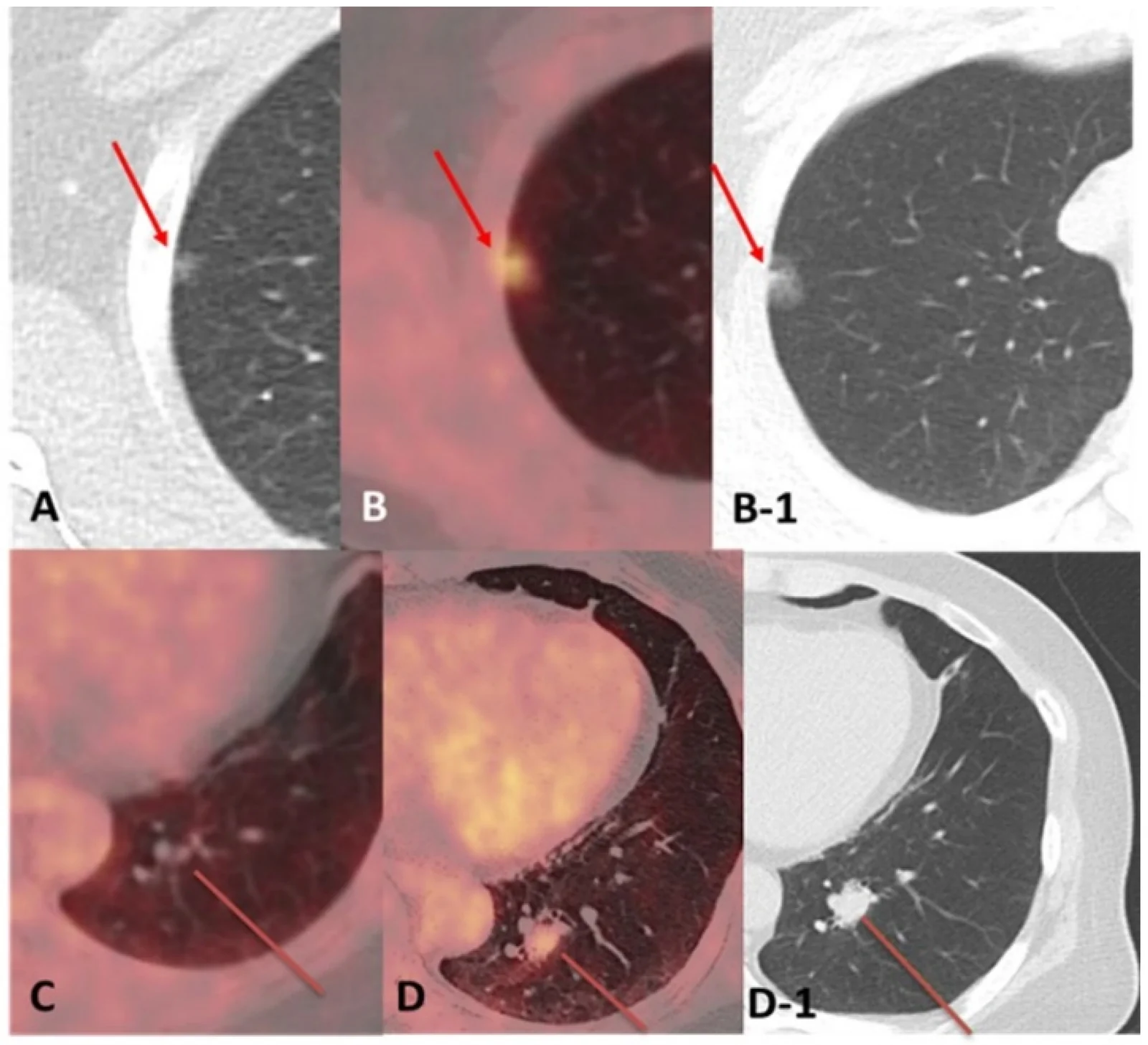
Ground-glass nodule (GGN) as atypical metastasis. A man with a history of melanoma of the right great toe. A GGN in the right upper lobe ((**A**); arrow) increased in size on follow-up imaging 1 year later ((**B**,**B-1**); arrows) and was surgically resected. Histopathologic examination demonstrated malignant melanoma, favoring pulmonary metastasis. Tumor cells partly grew along preserved alveolar structures, mimicking a lepidic growth pattern. A solid nodule in the left lower lobe ((**C**); line) showed interval growth on follow-up imaging 18 months later ((**D**,**D-1**); lines) and was also surgically resected and histologically confirmed as metastatic malignant melanoma.

Melanoma metastasis presenting as a pulmonary GGN is uncommon but has been reported [37,38]. Its frequency has since been quantified: among 87 patients with pulmonary metastases from malignant melanoma assessed on thin-section CT, GGNs were present in 13 (14.9%). Of the 11 patients with enlarging GGN metastases, 6 (54.5%) showed conversion to solid nodules. The mean tumor doubling time of GGNs, calculated in six patients, was 52.0 ± 33.5 days (range, 10.9–111 days), prompting the authors to recommend short-term follow-up at 1–2 months rather than the intervals applied to incidental nodules in the general population [39]. In the case shown in Figure 7, a GGN enlarged over one year and was confirmed as metastatic melanoma. Relatively rapid growth or the emergence of a solid component should therefore raise suspicion for metastatic disease, particularly when the lesion evolves concordantly with other metastases, even if FDG uptake remains low. A rapidly enlarging melanoma metastasis that initially appeared as a pure GGN and developed an eccentric solid component over 6 months showed no FDG uptake at that stage; resection demonstrated lepidic spread along the alveolar walls [40].

Conversely, a subsolid nodule may represent a primary lung adenocarcinoma rather than metastasis. In a series of 59 pathologically confirmed nodular ground-glass opacities in patients with extrapulmonary cancers, 40 (67.8%) were malignant and all of these were primary lung cancers, with none representing metastasis; metastases were present in the same cohort but appeared as solid nodules [41]. Alveolar-pattern metastasis is nevertheless well-documented. Among 76 patients with pulmonary metastases from pancreatic ductal adenocarcinoma, an alveolar growth pattern—ground-glass nodules, halo-sign nodules, air-space nodules, or consolidation—was predominant in 17 (22%). Histologic correlation in five patients with alveolar-pattern metastases demonstrated lepidic growth along preserved alveolar walls. In the overall cohort, six patients underwent resection because a solitary pulmonary lesion resembled a primary lung tumor [42].

**Figure 8:**
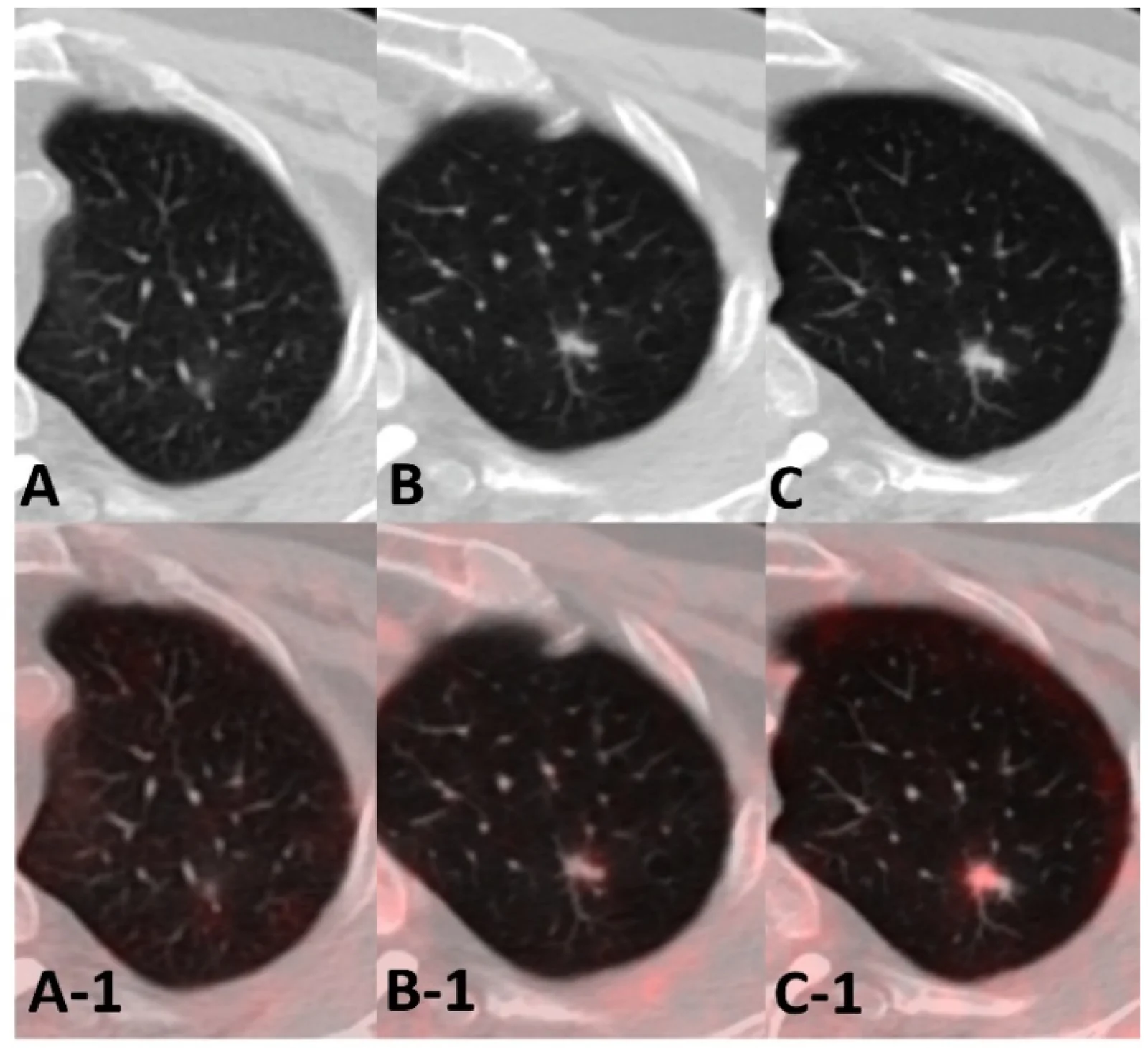
Subsolid nodule that proved to be a primary adenocarcinoma. A man under surveillance for lymphoma. (**A**,**A-1**) FDG PET/CT showed a small subsolid nodule in the left upper lobe with no discernible FDG uptake. (**B**,**B-1**) On follow-up after 2 years, a solid component and spiculated margins had developed, with only faint uptake (SUVmax 1.3). (**C**,**C-1**) After 4 years, focal uptake had become clearly visible (SUVmax 3.5) in the nodule, which had continued to enlarge. Left upper lobe bisegmentectomy revealed a moderately differentiated adenocarcinoma (acinar 60%, lepidic 40%; invasive component 0.6 cm of a 1.0 cm tumor, pT1aN0).

In the case shown in Figure 8, a subsolid nodule initially showed no discernible FDG uptake. Over the next 2 years, a solid component and spiculated margins developed, accompanied by only faint uptake (SUVmax 1.3); FDG uptake became clearly visible only after the lesion had enlarged further. Resection confirmed an adenocarcinoma with a 40% lepidic component and an invasive focus of 0.6 cm. These pathologic findings are consistent with low cellular density and a small metabolically active volume, which may account for the absent early uptake and susceptibility to partial-volume underestimation, as discussed in Section 2.1 and Section 2.3. The decisive findings were morphologic and temporal: an increasing solid proportion, emerging spiculation, and continued interval growth, all evident on the CT component before any clear rise in FDG uptake.

Teaching Point: In patients with cancer, a subsolid nodule may represent either an atypical metastasis or a primary lung adenocarcinoma, and both may initially show little or no FDG uptake. Morphologic evolution on serial CT may precede a clearly detectable increase in FDG uptake.

## 8. Future Directions

Several aspects of the interpretive framework outlined in this review require further validation. Respiratory-gated and deep-inspiration breath-hold acquisitions can reduce respiratory blurring and misregistration, but they are not routinely incorporated into whole-body oncologic protocols, and it remains unclear in which clinical settings their diagnostic benefit justifies the additional acquisition time. Whether automated nodule detection and classification tools can be validated in oncologic surveillance populations—where the task extends beyond binary benign–malignant classification to distinguishing metastasis, second primary lung cancer, infection, and other benign processes in patients who may harbor mixed etiologies—has yet to be established. Finally, no dedicated consensus guideline currently addresses incidentally detected pulmonary nodules in patients with known malignancy, a population excluded from the Fleischner Society recommendations [4]. Development and prospective validation of risk-adapted surveillance strategies incorporating index tumor type, lesion morphology, and longitudinal behavior therefore remain important areas for future investigation.

As one concrete example of such validation, prospective multicenter studies could test the hypothesis that lesion-level models integrating CT morphology, FDG avidity, primary tumor histology, and interval growth outperform metabolic assessment alone in discriminating malignant from benign pulmonary lesions detected on the CT component of oncologic FDG PET/CT. Such studies should enroll consecutive patients undergoing staging or surveillance PET/CT and prespecify the reference standard as histopathologic confirmation or, when pathology is unavailable, diagnosis based on protocol-defined longitudinal imaging criteria over a prespecified minimum follow-up interval. Reported outcomes should include diagnostic accuracy alongside clinically meaningful endpoints such as biopsy utilization, time to definitive diagnosis, and changes in clinical management attributable to pulmonary lesion characterization.

## 9. Conclusions

ILLs identified on the CT component of oncologic FDG PET/CT encompass a heterogeneous group of findings, including metastases, new primary lung cancers, inflammatory or infectious lesions, and mixed etiologies. Their incidental discovery or lack of corresponding FDG uptake should not be equated with clinical insignificance. As summarized in Table 1, these lesions require an integrated, lesion-by-lesion evaluation incorporating CT morphology, metabolic activity, interval evolution, and oncologic context. Subsolid or cavitary appearance, transient regression, or low FDG uptake should not lead to premature benign labeling. Recognition of atypical presentations—including PET-occult pulmonary metastases, metastases mimicking infection, metastatic subsolid nodules, new primary lung cancer, and the coexistence of infection and malignancy—is crucial for appropriate management.

This review is illustrative rather than epidemiologic: the cases were selected to demonstrate recurrent interpretive problems, and the relative frequency of these scenarios may vary across practice settings and according to regional disease prevalence. The clues summarized in Table 1 are intended as an expert synthesis to structure lesion-level reasoning, not as a validated decision rule or clinical guideline. The timing of CT surveillance, the indication for tissue confirmation, and, when a new primary lung cancer is suspected, the extent of staging work-up should be individualized in a multidisciplinary clinical setting, considering the index malignancy, the patient’s treatment status, and competing inflammatory or infectious diagnoses. The biologic and technical limitations outlined in Section 2 are fundamental to the interpretation of oncologic FDG PET/CT. Because PET findings should not be interpreted in isolation, meticulous review of the CT component—anchored to interval change on serial studies—is an integral part of the examination. When an ILL is small, subsolid, or metabolically inconspicuous, meticulous evaluation of the CT component and its interval evolution may provide the earliest—and sometimes the only—evidence of clinically significant pulmonary disease.

## Figures and Tables

**Table 1:** Diagnostic clues and suggested considerations for incidental lung lesions on the CT component of oncologic FDG PET/CT.

Clinical Scenario	CT Features	FDG Uptake	Malignancy Favored	Benign Favored	Suggested Diagnostic Consideration/Action *
PET-occult small metastasis	Solid subcentimeter nodule	Absent or faint	Interval growth; Primary tumor prone to pulmonary metastasis; Greater distance from pleura; Absence of an accompanying benign lesion	Long-term stability; Associated calcification; Typical perifissural morphology	Review thin-section lung window reconstruction; Compare with the prior CT component of PET/CT or diagnostic CT; If unavailable, obtain follow-up CT at a risk-adapted interval
Cavitary/subsolid metastasis mimicking infection	Multiple cavitary, irregular, or subsolid nodules	Mild	Primary tumor known to produce atypical pulmonary metastases (e.g., head and neck cancer, sarcoma)	Septic/fungal pattern with clinical signs of infection	Correlate with infection signs (fever, neutropenia, cultures); Reassess after treating suspected infection; Tissue confirmation for lesions that persist or grow discordantly
Inflammatory-appearing nodule	Irregular nodular opacity; May wax and wane	Variable; May be intense	Eventual growth despite transient regression	Complete and sustained resolution after appropriate therapy	Do not conclude that a lesion is benign based on a single transient decrease; Continue serial CT surveillance; Tissue confirmation if regrowth or emergence of aggressive imaging features
Coexisting TB/NTM with metastasis	Mixed nodules; Tree-in-bud, clustered/cavitary nodules	Variable	Single lesion with continuous growth among stable or waxing-and-waning lesions	Waxing-and-waning clustered nodules typical of NTM/TB	Assess each lesion separately (no single patient-level label); Flag the outlier lesion growing among waxing-and-waning nodules; Tissue confirmation of the discordant lesion
Synchronous or metachronous primary lung cancer	Solitary solid or predominantly solid nodule; Spiculation; Pleural retraction; Air bronchogram	Variable	Persistent growth independent of index cancer; Typical adenocarcinoma morphology	Long-term stability across serial studies; Complete and sustained resolution	Review prior CT for independent growth; Perform a lung cancer staging work-up with tissue confirmation
Subsolid nodule: metastasis versus primary lung adenocarcinoma	Pure ground-glass or part-solid nodule	Often absent or faint; May increase as the solid component enlarges	◆ Metastasis: Multiplicity; Change concordant with that of other known metastases; Melanoma or another index cancer with reported GGN-forming or alveolar-pattern metastases ◆ Primary adenocarcinoma: Solitary persistent lesion; Gradual enlargement; Increasing attenuation or solid component; Emerging spiculation or pleural retraction	Complete and sustained resolution with corresponding improvement in inflammatory clinical or imaging findings	Do not apply Fleischner criteria mechanically; Tailor CT follow-up interval to primary tumor and lesion growth kinetics; Tissue confirmation if growing or developing a solid component

* These suggestions represent a literature-informed expert synthesis rather than validated management recommendations.

## Data Availability

The data presented in this study are available on request from the corresponding author. The data are not publicly available due to patient privacy and ethical restrictions. All figures included in this manuscript were prepared by the authors using clinical imaging studies performed at Seoul St. Mary’s Hospital, College of Medicine, The Catholic University of Korea. The authors have the right to reproduce and publish all figures. None of the figures has been previously published in a peer-reviewed journal. All images were de-identified before inclusion, and their use was covered under the Institutional Review Board approval obtained for this review (KC26RISI0424).
